# Patients with Axial Spondyloarthritis Show an Altered Flexion/Relaxation Phenomenon

**DOI:** 10.3390/diagnostics11050810

**Published:** 2021-04-29

**Authors:** I. Concepción Aranda-Valera, Juan Luis Garrido-Castro, Alfonso Martínez-Galisteo, José Peña-Amaro, Cristina González-Navas, Antonio Cuesta-Vargas, Luis Jiménez-Reina, Eduardo Collantes-Estévez, Clementina López-Medina

**Affiliations:** 1 Rheumatology Department, Reina Sofía University Hospital, 14004 Cordoba, Spain; conchita_87_8@hotmail.com (I.C.A.-V.); edcollantes@yahoo.es (E.C.-E.); clementinalopezmedina@gmail.com (C.L.-M.); 2 G05 Group, Maimonides Institute of Biomedical Research of Cordoba (IMIBIC), 14004 Cordoba, Spain; crsgonzaleznavas@yahoo.es; 3 University of Cordoba, 14004 Cordoba, Spain; an1magaa@uco.es (A.M.-G.); cm1peamj@gmail.com (J.P.-A.); cm1jirel@uco.es (L.J.-R.); 4 Malaga Institute of Biomedical Research (IBIMA), University of Malaga, 29016 Malaga, Spain; acuesta@uma.es

**Keywords:** axial spondyloarthritis, surface electromyography (sEMG), flexion relaxation ratio, functional assessment, clinimetric properties

## Abstract

Axial spondyloarthritis (axSpA) is a chronic rheumatic disease characterized by the presence of inflammatory back pain. In patients with chronic low back pain, the lumbar flexion relaxation phenomenon measured by surface electromyography (sEMG) differs from that in healthy individuals. However, sEMG activity in axSpA patients has not been studied. The purpose of this study was to analyze the flexion relaxation phenomenon in axSpA patients. A study evaluating 39 axSpA patients and 35 healthy controls was conducted. sEMG activity at the erector spinae muscles was measured during lumbar full flexion movements. sEMG activity was compared between axSpA patients and the controls, as well as between active (BASDAI ≥ 4) and non-active (BASDAI < 4) patients. The reliability (using intraclass correlation coefficients (ICC)), criterion validity and discriminant validity using the area Under the curve (AUC) for the inverse flexion/relaxation ratio (1/FRR) were evaluated. Significant differences (*p* < 0.05) were observed between axSpA patients and the control group in lumbar electric activity, especially during flexion, relaxation, and extension and in FRR and 1/FRR (0.66 ± 0.39 vs. 0.25 ± 0.19, respectively). In addition, significant differences were found between active and non-active but also between non-active and healthy subjects. The sEMG showed good reliability (ICC > 0.8 for 1/FRR) and criterion validity. ROC analysis showed good discriminant validity for axSpA patients (AUC = 0.835) vs. the control group using 1/FRR. An abnormal flexion/relaxation phenomenon exists in axSpA patients compared with controls. sEMG could be an additional objective tool in the evaluation of patient function and disease activity status.

## 1. Introduction

Axial spondyloarthritis (axSpA) is a chronic, inflammatory, rheumatic disease with high phenotypic heterogeneity. It is characterized by new bone formation in the sacroiliac joints and axial skeleton. Inflammatory back pain (IBP) represents a clinical expression of lumbar spine inflammation, which leads to structural damage and a decrease in spinal mobility [1,2].

The concept of mechanical stress in the pathogenesis of axSpA has recently been revitalized, with the theory that interactions between biomechanical factors and the innate immune response may lead to the development of enthesitis [3,4]. This activation of metabolic pathways and cytokines would not be confined exclusively to entheses but would also involve tissues immediately adjacent to this organ: bone, fascia, extra enthesis, and the Synovio-Entheseal Complex (SEC) [5]. Some authors have reported pathological changes in the paravertebral muscles in axSpA patients (such as muscle fiber atrophy and cytoarchitectural abnormalities) [6,7], which could be associated with overactivity through their paravertebral muscles and altered load-sharing capability of the tissues [8]. These findings suggest that hypertonicity in the axSpA could involve an excess of joint forces associated with damage to the vertebral enthesis and raise the issue of whether this phenomenon is a cause or consequence of pathway activation and, therefore, of structural damage in axSpA patients [6,9].

Surface electromyography (sEMG) has been suggested as a useful objective tool in the assessment of musculoskeletal dysfunction associated with mechanical low back pain (LBP) [10,11]. The dynamic measurement of the sEMG activity in paraspinal muscles can be useful in differentiating between patients with LBP and asymptomatic subjects and to detect changes after treatment [12]. In maximum voluntary flexion (MVF), sEMG activity is often at or below the level of sEMG activity during standing [13]. However, in people with LBP, this paraspinal relaxation tends to be absent or decreased [14,15]. The flexion/relaxation (F/R) phenomenon is important because it enables the full expression of lumbar flexion to occur in normal subjects [16]. sEMG allows both a patient and clinician to have direct and immediate access to muscle functioning that is not possible with manual palpation or visual observation [17]. A common factor used to evaluate the F/R phenomenon is the F/R ratio (FRR), as well as the inverse FRR (1/FRR) [18]. The latter is essentially the percentage to which the lumbar muscles become electrically silent during full flexion in comparison with the higher activity seen during forward flexion [18]. However, to our knowledge, the FRR has not been explored in axSpA patients. On the basis of the foregoing, the analysis of sEMG in the paravertebral musculature could be of interest due to its possible association with biomechanical stress and motor control in axSpA patients. Thus, in this study, we propose the first clinometric approach to sEMG activity in these patients, focusing on the FRR in axSpA patients and clinimetric properties with three goals: (a) to describe the activity and variability of sEMG in patients with axSpA; (b) to demonstrate the reliability of sEMG in axSpA patients and healthy subjects; and (c) to evaluate the validity of sEMG to distinguish not only between axSpA patients vs. healthy subjects but also between axSpA active patients vs. non-active patients.

## 2. Materials and Methods

### 2.1. Patients

A total of 39 patients with axSpA, as determined by the Assessment of Spondyoarthritis International Society (ASAS) criteria [19], and 35 healthy controls were included in the study. Inclusion criteria for the patient group were as follows: (a) patients ages ≥ 18 years with a clinical diagnosis of adult-onset axSpA of ≥3 months duration and (b) patients who met the ASAS classification criteria. Inclusion criteria for healthy controls were: (a) ages ≥ 18 years and (b) absence of LBP or IBP. Patients suffering from disc disease or who had undergone previous surgery were excluded from the two groups.

Eligible participants (axSpA patients and controls) were scheduled for a physical examination, in which they completed questionnaires, were screened by study physicians, and underwent electromyography study.

All patients signed a consent form, and the protocol was approved by the “Hospital Universitario Reina Sofía” Ethics Committee (Ref. 1393-N-16).

### 2.2. EMG Recordings

A surface electromyogram (sEMG) telemetry system (TELEMYO 2400T^®®^; Noraxon USA Inc., 13,430 N. Scottsdale Rd., Suite 104, Scottsdale, AZ 85254, USA) was used. A synchronised video recording (25 Hz) was performed using a video camera (SONY handycam DCR-HC23, Tokyo, Japan). The video was used to distinguish events in the sEMG signal.

**Figure 1:**
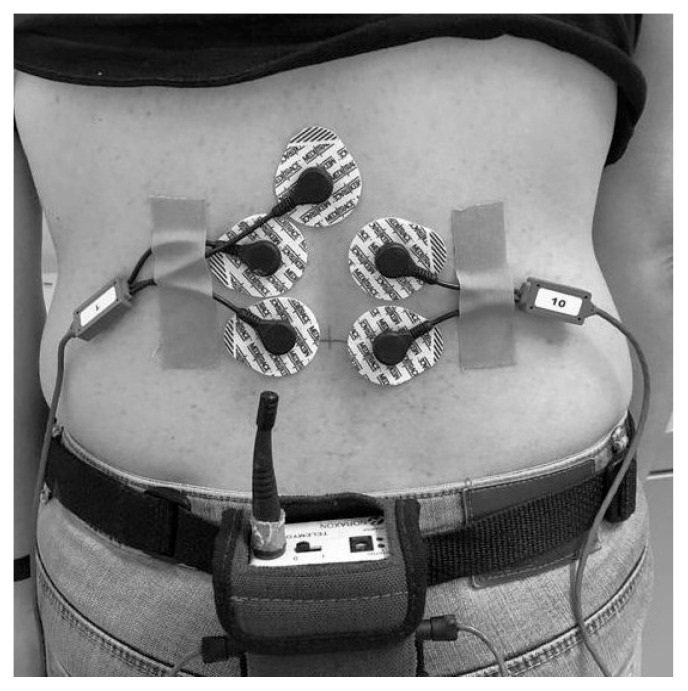
Electrodes placement.

The methodology for the F/R test was based on the work of Watson et al. [14] Electrodes were placed paraspinally (right and left lumbar erector spinae) at the L4–L5 level and separated at 2.5 cm from the spinous process. The reference electrode was placed on the spinous process at the L3 level, and the sensors were oriented so that they were parallel to the muscle fibers (Figure 1). The skin underlying the electrode was cleaned with cotton soaked in alcohol to provide a better conductivity.

Participants were instructed to move from a standing position to full frontal flexion in a gentle manner for 10 s. Full flexion was maintained for 5 s, followed by a return to the vertical position for another 10 s. After a rest of 5 s, the complete movement was repeated. Two cycles were recorded to calculate variability between measurements.

### 2.3. Data Reduction

Prior to study and interpretation, the electromyographic signals were processed (Noraxon Myoresearch^®®^ XP, Noraxon USA, Scottsdale, AZ, USA), applying some filters: rectification, smoothing (RMS-500 ms window), and finally a 10 Hz Butterworth low-pass filter. The sEMG signal was divided into phases based on the time points identified in the channel position data. The phases were identified as standing, flexion, relaxation, and extension.

### 2.4. Variables

**Figure 2:**
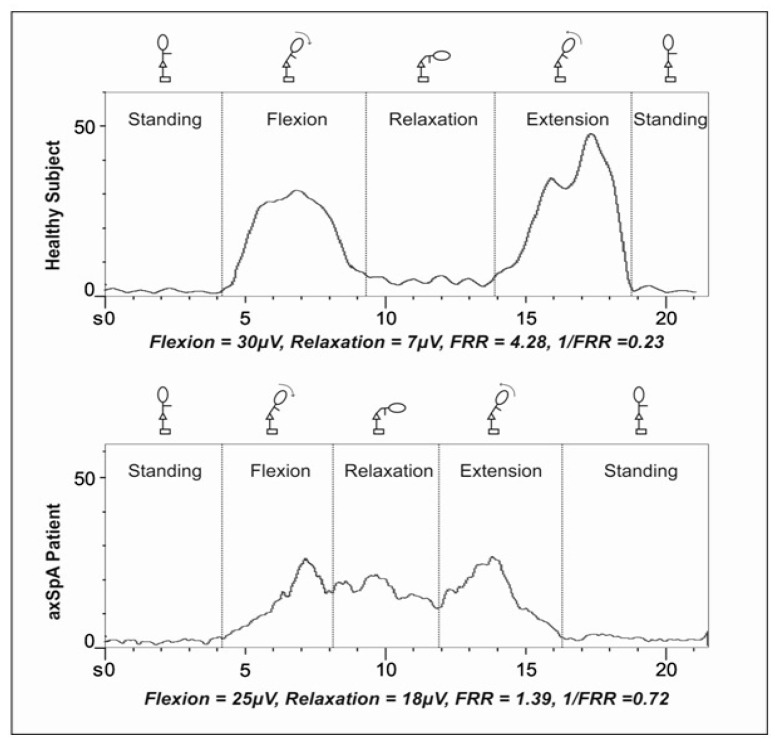
sEMG activity and results obtained by a healthy subject and an axSpA patient.

Sociodemographic (age, sex) and anthropometric data (weight, height, and body mass index (BMI)) were collected for both groups (axSpA patients and controls). All subjects also underwent sEMG. During sEMG, the patient started in a standing position, and he/she performed a sequence of a flexion movements, relaxation (or full flexion), extension, and a return to standing (Figure 2).

The lumbar muscle electric activity measured in μV was obtained in each phase. In addition, the FRR was calculated considering the maximum value of sEMG during flexion divided by the value during relaxation (full flexion). The inverse FRR (1/FRR) was also calculated, which has the advantage of providing a normalised sEMG factor, which makes it possible to compare sEMG factors over time and across individuals [14,18]. Values for 1/FRR typically range from 0 to 1 since sEMG activity is normally lower during relaxation (full flexion) than during flexion movement. When 1/FRR is 1, sEMG activity during flexion and relaxation would be the same (no silence at all). Figure 2 shows an example of sEMG activity in each phase of the movement for an individual healthy subject and a patient. In a healthy subject, the typical pattern shows high electric activity during flexion, a silent phase during relaxation or full flexion, and high electric activity during extension, with a 1/FRR near 0. In case of absence of silent phase, the 1/FRR would be near 1.

Four variables were completed by the axSpA group, which served as criterion validity: function index was measured with the Bath Ankylosing Spondylitis Functionality Index (BASFI) [20]; disease activity was measured with the Bath Ankylosing Spondylitis Disease Activity Index (BASDAI) [21]. Mobility was defined according to J. Sieper’s review [2]: cervical rotation, tragus-wall distance, lateral spinal flexion, modified Schöber test, intermaleolar distance and the Bath Ankylosing Spondylitis Metrology Index (BASMI) [22].

A rheumatologist (I.C. AV) experienced in the use of the sEMG device and in conventional metrology performed the entire patient measurement process.

### 2.5. Statistical Analysis

The sample size estimation was calculated so that mean effect sizes of 0.3 could be detected with a power of 80% and a risk α of 5%.

Descriptive data are presented as the mean ± standard deviation (SD) for qualitative variables and as frequencies and percentages for qualitative variables. A *p*-value < 0.05 was considered significant, and the statistical analysis was performed using IBM SPSS software (version 17.0) and R statistical language R Studio (version 1.1.383).

First, demographic and anthropometric data between axSpA patients and the healthy group were compared to verify that both groups were similar.

Second, the average values of sEMG measurements were compared between axSpA patients vs. the control group, between active axSpA vs. non-active axSpA (defining active and non-active patients as a BASDAI ≥ 4 or BASDAI < 4, respectively), and between non-active axSpA vs. controls by using a Student’s *t* test for independent samples.

The clinimetric proprieties of the 1/FRR were evaluated according the COnsensus-based Standards for the Selection of health status Measurement INstruments (COSMIN) [23].

#### 2.5.1. Reliability

The internal consistency of the measurements was evaluated in all patients through the use of an intraclass correlation coefficient (ICC). Measurement errors were calculated using standard deviation.

#### 2.5.2. Criterion Validity

To determine factors associated with sEMG in axSpA patients, Pearson’s linear correlations were performed between sEMG data and conventional scores, BASDAI, BASFI, and BASMI.

#### 2.5.3. Discriminant Validity

Four receiver operating characteristic (ROC) curves analyses (axSpA vs. controls, active axSpA vs. non-active axSpA, non-active axSpA vs. controls and active axSpA vs. controls) evaluated the validity of 1/FRR to distinguish between axSpA patients and healthy subjects and between axSpA active patients vs. non-active patients.

## 3. Results

Among the 74 subjects (39 axSpA and 35 healthy) included in the study, 56 (75.7%) were men, and the average age was 44 ± 10.2 years (Table 1). There were no significant differences between the groups in terms of age, sex, weight, height, or BMI (Table 1).

### 3.1. sEMG Measurements between axSpA and Controls

No significant differences appeared between the right nor left sides of the sEMG measurements, so mean values were considered for the analysis (data not shown).

Table 2 shows the average values in μV of each sEMG measure. Significantly reduced electric activity was observed between axSpA patients vs. the control group during flexion (20.38 ± 11.62 vs. 36.50 ± 20.09) and extension (39.07 ± 23.45 vs. 66.09 ± 15.53), and increased electric activity was observed during relaxation (or full flexion) (13.08 ± 11.69 vs. 6.87 ± 4.02). In addition, a reduced FRR was found among axSpA vs. controls (2.40 ± 1.89 vs. 7.13 ± 6.64), meaning that the electric activity during flexion and relaxation was similar among axSpA patients and that controls had a decrease in electric activity during full flexion (silent phase). Similarly, an increased 1/FRR was found in axSpA patients vs. controls (0.66 ± 0.39 vs. 0.25 ± 0.19).

### 3.2. sEMG Measurements between Active axSpA and Non-Active axSpA

When patients were grouped into active axSpA (BASDAI ≥ 4, *n* = 22) vs. non-active axSpA (BASDAI < 4, *n* = 17) (Table 2), we found that active patients showed lower values of EMG signals in standing (6.59 ± 2.18 vs. 10.47 ± 5.19) and extension (23.38 ± 12.40 vs. 46.49 ± 23.28), lower FRR (1.51 ± 1.05 vs. 3.11 ± 2.11), and a higher score in 1/FRR (0.82 ± 0.31 vs. 0.57 ± 0.42) against non-active axSpA patients. We also compared non-active axSpA patients vs. the control group, and we found a significant decrease in electric activity in flexion and extension, a lower FRR and a higher 1/FRR in non-active axSpA patients.

### 3.3. Reliability

The reproducibility of these measurements was evaluated in all patients with the ICCs (Table 2). Standing, flexion, extension and 1/FRR measures showed excellent interrater agreement (ICC > 0.8), while relaxation and FRR showed good agreement (ICC > 0.6).

### 3.4. Criterion Validity

Pearson correlations for age, function, disease activity and mobility in the axSpA group are shown in Table 3. Index 1/FRR showed a strong negative linear relationship with lateral flexion (r = −0.71), a moderate negative correlation with the Schöber measure (r = −0.55), and a moderate positive linear correlation with the BASFI (r = 0.52) and BASMI (r = 0.65). These results showed that an increment in 1/FRR (i.e., absence of silent phase during relaxation or similar electric activity during flexion and relaxation) in axSpA patients is associated with poorer mobility (i.e., less lateral flexion, less Schober, and higher BASMI) as well as poorer function (higher BASFI).

### 3.5. Discriminant Validity

**Figure 3:**
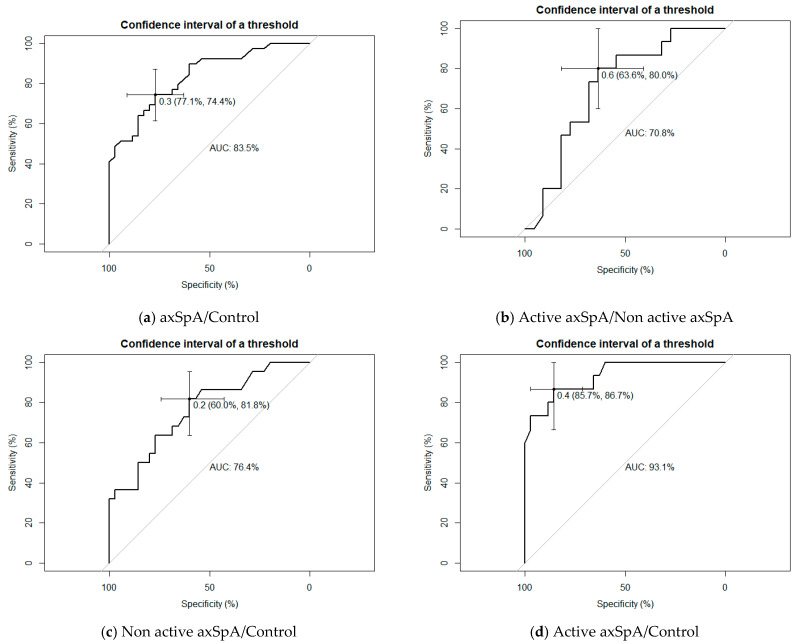
ROC analysis using 1/FRR to distinguish axSpA patients and healthy controls.

Figure 3 shows results of the four ROC analyses (axSpA vs. controls, active axSpA vs. non-active axSpA, non-active axSpA vs. controls and active axSpA vs. controls) regarding 1/FRR. A cut-off of 0.3 in the 1/FRR measure revealed an AUC of 0.835 when comparing axSpA patients vs. the control group, with a sensitivity of 77.1% and a specificity of 74.4% (Figure 3a). This index also produced useful results for distinguishing between active and non-active axSpA (AUC = 0.708) and between non-active axSpA and controls (AUC = 0.764), and especially between active axSpA and controls (AUC = 0.931) (Figure 3b–d).

## 4. Discussion

To our knowledge, this is one of the first studies aiming to evaluate flexion/relaxation phenomena in axSpA patients. Our results highlight that an abnormal flexion/relaxation phenomenon (measured by FRR and 1/FRR) exists in axSpA patients compared with control subjects, suggesting the absence of a silent phase during relaxation or similar electric activity during flexion and relaxation in these patients.

In our study, the FRR in axSpA patients was similar to that found by Watson et al. [14] in a sample of chronic LBP patients. This F/R phenomenon and its subsequent reduction in FRR in LBP patients have been described in the literature. Geisser et al. [24] found a relationship between fear of movement in the context of pain and loss of flexion relaxation in LBP patients, and other authors explained trunk motor control and its dysfunction in patients with LBP using sEMG [25,26]. A loss of F/R might also contribute to the conversion of back pain from acute to chronic. When muscles cannot relax normally, they will fatigue more quickly, leading to co-contraction of other trunk muscles to help maintain spinal stability [27]. This could be justified by possible changes in the predominance of muscle fibre types in the lumbar region; in this sense, it has been reported that chronic low back pain produces a conversion of type 1 muscle fiber to type 2 that are more fatigued [7].

It has been suggested that paravertebral muscle atrophy and fibrosis in axSpA are the final consequences of the progressive disuse secondary to axial joint dysfunction caused by arthrodesis and spinal ankylosis [28]. However, another phenomenon could be added to this muscle involvement in axSpA. In muscle biopsies of patients with axSpA, atrophy, fibrosis and pathological cytoarchitectural changes in muscle fibers (core, multicore, core-targetoid, and moth-eaten) occur [6,29]. Although these changes in muscle pathology are usually nonspecific, they occur experimentally after tenotomy and are interpreted as the adaptative response of muscle fibers to their shortening in length as a consequence of tendon injury [30,31]. In addition, a reduction in the size of the muscle fibers occurs together with an increase in connective tissue, which is understood by the structural continuity of the extracellular matrix or muscular connective tissue with the tendon and the periosteum known as the fascial system. This system seems to act in an integrated way; therefore, an injury produced in a given territory can generate an adaptive or pathological response in a related structure not limited to the injured tissue [32,33]. This structural response would justify the rigidity of muscles in the axSpA patients and the sEMG results. In this type of study, it is common to refer to the area when we measure the sEMG. We say that we measure the activity of erector spinae, although it is a combination of three muscles (iliocostal, longissimus y spinalis) that cannot completely separated.

In this analysis, we also confirmed the reliability and concordance of sEMG measures (especially with the 1/FRR index), not only in axSpA patients but also in healthy subjects. We also demonstrated that the variability of FRR and 1/FRR in axSpA patients is directly associated with disease activity, functionality, and mobility, as measured by BASDAI, BASFI and BASMI, respectively. This prompted us to think that the disease status and physical condition of the patient could act as a cause or consequence for the loss of the F/R phenomenon, which could be demonstrated with further longitudinal analysis. This alteration in the F/R phenomenon has also been associated with LBP disability scores in previous studies [14,18,31].

Regarding the validity of the sEMG, our study shows that a value of 0.3 in 1/FRR has the predictive ability to discriminate axSpA patients from normal subjects, indicating that an important alteration in EMG activity exists in these patients. Interestingly, a good AUC was also found when comparing active vs. non-active axSpA patients, which means that this could be an additional tool to evaluate disease activity in patients. Finally, as expected, the greatest AUC was observed between active axSpA patients vs. the control group, i.e., between patients with high levels of inflammation and pain and healthy subjects.

Our study has some limitations but also several strengths. One limitation is that we did not include patients with mechanical LBP to be compared with axSpA patients. However, this was not the goal of our study. We conducted a first approach in axSpA patients, not patients in the whole group of spine diseases, even though this studies comparing sEMG in IBP patients against mechanical LBP are ongoing in our department. The sensitivity and specificity of sEMG could be increased by using multiple measures. Although some authors indicate that sEMG is not usable in daily clinical practice (especially in the field of neurology) [34], many others currently contradict this statement [35,36] as sensor technology advances. Further research is needed to determine the combination of measures that are cost-effective and prospectively validated as a classification scheme. Another limitation is the small sample size included in our study. However, this was calculated during the project design with a sufficient power to detect differences between groups. The main strength of this study is that it is the first to evaluate sEMG activity in axSpA patients; thus, this could be the first step in the evaluation of sEMG and hypertonicity in this pathology.

## 5. Conclusions

This study demonstrates that an abnormal flexion/relaxation phenomenon exists in axSpA patients and that sEMG could be an additional objective tool in the evaluation of patient functionality and disease activity status.

## Figures and Tables

**Table 1:** Demographics data in both groups.

Demographic Data	axSpA Group *n* = 39	Control Group *n* = 35	*p*
Age (±SD) years	46 (8.06)	42 (11.89)	0.115
Sex (%) men	29 (74.4%)	27 (77.1%)	0.780
women	10 (25.6%)	8 (22.9%)	
Weight (±SD) kg	80.4 (17.2)	78.9 (16.1)	0.702
Height (±SD) m	1.7 (0.6)	1.7 (0.8)	0.197
BMI (SD)	27.6 (5.8)	26.1 (3.8)	0.211

BMI: Body Mass Index. SD: Standard Deviation.

**Table 2:** Average values in each phase by group, significant differences, and reliability results.

	axSpA *n* = 39	Control *n* = 35	*p* (1)	Active axSpA *n* = 22	Non Active axSpA *n* = 17	*p* (2)	*p* (3)	ICC
FRR	2.40 (1.89)	7.13 (6.64)	***	1.51 (1.05)	3.11 (2.11)	**	**	0.682
1/FRR	0.66 (0.39)	0.25 (0.19)	***	0.82 (0.31)	0.57 (0.42)	**	**	0.938
Flx/Ext	0.60 (0.32)	0.59 (0.39)	N.S.	0.67 (0.30)	0.57 (0.34)	N.S.	N.S.	0.560
Standing	9.35 (4.92)	9.57 (4.20)	N.S.	6.59 (2.18)	10.47 (5.19)	**	N.S.	0.840
Flexion	20.38 (11.62)	36.50 (20.09)	***	16.96 (10.86)	21.20 (11.37)	N.S.	***	0.817
Relaxation	13.08 (11.69)	6.87 (4.02)	**	14.60 (11.59)	10.54 (10.94)	N.S.	N.S.	0.631
Extension	39.07 (23.45)	66.09 (25.53)	***	23.48 (12.40)	46.49 (23.28)	***	**	0.927

Mean values (SD) of EMG signals in μV. FRR: flexion-relaxation ratio; 1/FRR: inverse flexion-relaxation ratio; Flx/Ext: flexion-relaxation index. Active axSpA: BASDAI >= 4. Non Active axSpA: BASDAI < 4. Student *t* test Differences: (1) axSpA/Control (2) Active/Non Active (3) Non Active/Control. ** *p* < 0.01; *** *p* < 0.001, N.S.: Not significant.

**Table 3:** Correlations between sEMG measures and other variables for axSpA group.

	Age	Lat. Flex.	Schober	Cerv. Rot.	BASDAI	BASFI	BASMI
FRR	−0.19	0.60 ***	0.53 ***	0.26	−0.39 *	−0.44 **	−0.59 ***
1/FRR	0.27	−0.71 ***	−0.55 ***	−0.27	0.38 *	0.52 ***	0.65 ***
Flx/Ext	0.05	−0.40 *	−0.45 **	0	0.19	0.12	0.39 *
Standing	0.02	0.16	−0.1	0.17	−0.35 *	−0.23	−0.15
Flexion	−0.01	0.24	−0.08	0.18	−0.26	−0.28	−0.18
Relaxation	0.18	−0.24	−0.40 *	−0.1	0.14	0.15	0.29
Extension	−0.1	0.60 ***	0.31	0.43 **	−0.50 **	−0.47 **	−0.60 ***

FRR: flexion-relaxation ratio; 1/FRR: inverse flexion-relaxation ratio; Flx/Ext: flexion-relaxation index. * *p* < 0.05; ** *p* < 0.01; *** *p* < 0.001.

## Data Availability

The data presented in this study are available on request from the corresponding author.

## References

[1] Sieper J., Rudwaleit M., Baraliakos X., Brandt J., Braun J., Burgos-Vargas R., Dougados M., Hermann K.-G., Landewé R., Maksymowych W. (2009). The Assessment of SpondyloArthritis international Society (ASAS) handbook: A guide to assess spondyloarthritis. Ann. Rheum. Dis..

[2] Sieper J., van der Heijde D., Landewé R., Brandt J., Burgos-Vagas R., Collantes-Estevez E., Dijkmans B., Dougados M., Khan M.A., Leirisalo-Repo M. (2009). New criteria for inflammatory back pain in patients with chronic back pain: A real patient exercise by experts from the Assessment of SpondyloArthritis international Society (ASAS). Ann. Rheum. Dis..

[3] McGonagle D., Stockwin L., Isaacs J., Emery P. (2001). An enthesitis based model for the pathogenesis of spondyloarthropathy. additive effects of microbial adjuvant and biomechanical factors at disease sites. J. Rheumatol..

[4] Jacques P., Lambrecht S., Verheugen E., Pauwels E., Kollias G., Armaka M., Verhoye M., Van Der Linden A., Achten R., Lories R.J. (2014). Proof of concept: Enthesitis and new bone formation in spondyloarthritis are driven by mechanical strain and stromal cells. Ann. Rheum. Dis..

[5] Lories R.J.U., Derese I., Luyten F.P. (2005). Modulation of bone morphogenetic protein signaling inhibits the onset and progression of ankylosing enthesitis. J. Clin. Investig..

[6] Masi A.T., Sierakowski S., Kim J.M. (2005). Jacques Forestier’s vanished bowstring sign in ankylosing spondylitis: A call to test its validity and possible relation to spinal myofascial hypertonicity. Clin. Exp. Rheumatol..

[7] Mannion A.F. (1997). Fibre type characteristics and function of the human paraspinal muscles: Normal values and changes in association with low back pain. J. Electromyogr. Kinesiol..

[8] Kim M., Yi C., Kwon O., Cho S., Cynn H., Kim Y., Hwang S.H., Choi B.R., Hong J.A., Jung D.H. (2013). Comparison of lumbopelvic rhythm and flexion-relaxation response between 2 different low back pain subtypes. Spine.

[9] Masi A.T. (2011). An added perspective on the 2009 SPARTAN and IGAS report: An innate axial myofascial hypertonicity. J. Rheumatol..

[10] Dankaerts W., O’Sullivan P., Burnett A., Straker L., Davey P., Gupta R. (2009). Discriminating healthy controls and two clinical subgroups of nonspecific chronic low back pain patients using trunk muscle activation and lumbosacral kinematics of postures and movements: A statistical classification model. Spine.

[11] Neblett R., Brede E., Mayer T.G., Gatchel R.J. (2013). What is the best surface EMG measure of lumbar flexion-relaxation for distinguishing chronic low back pain patients from pain-free controls?. Clin. J. Pain.

[12] Neblett R., Mayer T.G., Brede E., Gatchel R.J. (2010). Correcting abnormal flexion-relaxation in chronic lumbar pain: Responsiveness to a new biofeedback training protocol. Clin. J. Pain.

[13] Geisser M.E., Ranavaya M., Haig A.J., Roth R.S., Zucker R., Ambroz C., Caruso M. (2005). A Meta-Analytic Review of Surface Electromyography Among Persons with Low Back Pain and Normal, Healthy Controls. J. Pain.

[14] Watson P.J., Booker C.K., Main C.J., Chen A.C.N. (1997). Surface electromyography in the identification of chronic low back pain patients: The development of the flexion relaxation ratio. Clin. Biomech..

[15] Ambroz C., Scott A., Ambroz A., Talbott E.O. (2000). Chronic low back pain assessment using surface electromyography. J. Occup. Environ. Med..

[16] Neblett R., Mayer T.G., Brede E., Gatchel R.J. (2014). The effect of prior lumbar surgeries on the flexion relaxation phenomenon and its responsiveness to rehabilitative treatment. Spine J..

[17] Neblett R. (2016). Surface Electromyographic (SEMG) Biofeedback for Chronic Low Back Pain. Healthcare.

[18] Neblett R., Mayer T.G., Gatchel R.J., Keeley J., Proctor T., Anagnostis C. (2003). Quantifying the lumbar flexion-relaxation phenomenon: Theory, normative data, and clinical applications. Spine.

[19] Rudwaleit M., van der Heijde D., Landewé R., Listing J., Akkoc N., Brandt J., Braun J., Chou C.T., Collantes-Estevez E., Dougados M. (2009). The development of assessment of spondyloarthritis international society classification criteria for axial spondyloarthritis (part II): Validation and final selection. Ann. Rheum. Dis..

[20] Calin A., Garrett S., Whitelock H., Kennedy L.G., O’Hea J., Mallorie P., Jenkinson T. (1994). A new approach to defining functional ability in ankylosing spondylitis: The development of the Bath Ankylosing Spondylitis Functional Index. J. Rheumatol..

[21] Garrett S., Jenkinson T., Kennedy L.G., Whitelock H., Gaisford P., Calin A. (1994). A new approach to defining disease status in ankylosing spondylitis: The Bath Ankylosing Spondylitis Disease Activity Index. J. Rheumatol..

[22] van der Heijde D., Landewé R., Feldtkeller E. (2008). Proposal of a linear definition of the Bath Ankylosing Spondylitis Metrology Index (BASMI) and comparison with the 2-step and 10-step definitions. Ann. Rheum. Dis..

[23] Mokkink L.B., Terwee C.B., Patrick D.L., Alonso J., Stratford P.W., Knol D.L., Bouter L.M., de Vet H.C. (2010). The COSMIN study reached international consensus on taxonomy, terminology, and definitions of measurement properties for health-related patient-reported outcomes. J. Clin. Epidemiol..

[24] Geisser M.E., Haig A.J., Wallbom A.S., Wiggert E.A. (2004). 400 Pain-related fear, lumbar flexion, and dynamic EMG among persons with chronic musculoskeletal low back pain. Clin. J. Pain.

[25] Boucher J.A., Preuss R., Henry S.M., Nugent M., Larivière C. (2018). Trunk postural adjustments: Medium-term reliability and correlation with changes of clinical outcomes following an 8-week lumbar stabilization exercise program. J. Electromyogr. Kinesiol..

[26] Russo M., Deckers K., Eldabe S., Kiesel K., Gilligan C., Vieceli J., Crosby P. (2018). Muscle Control and Non-specific Chronic Low Back Pain. Neuromodulation.

[27] Granata K.P., Marras W.S. (2000). Cost-benefit of muscle cocontraction in protecting against spinal instability. Spine.

[28] Ozturk E.C., Yagci I. (2021). The structural, functional and electrophysiological assessment of paraspinal musculature of patients with ankylosing spondylitis and non-radiographic axial spondyloarthropathy. Rheumatol. Int..

[29] Zhang Y., Xu H., Hu X., Zhang C., Chu T., Zhou Y. (2016). Histopathological changes in supraspinous ligaments, ligamentum flava and paraspinal muscle tissues of patients with ankylosing spondylitis. Int. J. Rheum. Dis..

[30] Pena-Amaro J., Luque E., Jimena I., Noguera F., Castilla S., Vaamonde R. (2019). Abnormalities in tenectomized muscle fiber repair. Eur. J. Anat..

[31] Jamali A.A., Afshar P., Abrams R.A., Lieber R.L. (2000). Skeletal muscle response to tenotomy. Muscle Nerve.

[32] Zügel M., Maganaris C.N., Wilke J., Jurkat-Rott K., Klingler W., Wearing S.C., Findley T., Barbe M.F., Steinacker J.M., Vleeming A. (2018). Fascial tissue research in sports medicine: From molecules to tissue adaptation, injury and diagnostics: Consensus statement. Br. J. Sports Med..

[33] Csapo R., Gumpenberger M., Wessner B. (2020). Skeletal Muscle Extracellular Matrix–What Do We Know About Its Composition, Regulation, and Physiological Roles? A Narrative Review. Front. Physiol..

[34] Pullman S.L., Goodin D.S., Marquinez A.I., Tabbal S., Rubin M. (2000). Clinical utility of surface EMG Report of the Therapeutics and Technology Assessment Subcommittee of the American Academy of Neurology. Neurology.

[35] Zwarts M.J., Stegeman D.F. (2003). Multichannel surface EMG: Basic aspects and clinical utility. Muscle Nerve.

[36] Mohseni Bandpei M.A., Rahmani N., Majdoleslam B., Abdollahi I., Ali S.S., Ahmad A. (2014). Reliability of surface electromyography in the assessment of paraspinal muscle fatigue: An updated systematic review. J. Manip. Physiol. Ther..

